# Non-inferiority comparative clinical trial between early oral REFEEDING and usual oral REFEEDING in predicted mild acute biliary pancreatitis

**DOI:** 10.1186/s12876-020-01363-3

**Published:** 2020-07-16

**Authors:** Edgard Efrén Lozada-Hernández, Omar Barrón-González, Santa Vázquez-Romero, Martin Cano-Rosas, Evelia Apolinar-Jimenez

**Affiliations:** 1 Department of Surgery and Clinical Research, Hospital Regional de Alta Especialidad del Bajío, Circuito Quinta los Naranjos # 145 B. Colonia Quinta los Naranjos, León, Guanajuato Mexico; 2 Department of Surgery, Unidad Médica de Alta Especialidad Bajío, Instituto Mexicano del Seguro Social, León, Guanajuato Mexico; 3 Department of Clinical Nutrition, Hospital Regional de Alta Especialidad del Bajío, León, Guanajuato Mexico

**Keywords:** Mild acute biliary pancreatitis, Oral feeding, Non-inferiority clinical trial, Nil per os

## Abstract

**Background:**

The aim of the study was to compare the onset of oral feeding in the first 24 h after hospital admission with usual oral refeeding and determine whether the timing of the onset of oral feeding influences the recurrence of pain or alters the blood levels of pancreatic enzymes in patients with predicted mild acute biliary pancreatitis.

**Methods:**

This non-inferiority randomized controlled trial was carried out between September 2018 and June 2019 after receiving authorization from the ethics committee for health research. Patients with a diagnosis of predicted mild acute biliary pancreatitis were divided into Group A (early oral refeeding, EOR) and Group B (usual oral refeeding, UOR). Outcome measures included pancreatic lipase levels, the systemic inflammatory response (concentrations of leukocytes), feasibility (evaluated by abdominal pain recurrence), the presence and recurrence of gastrointestinal symptoms and the length of hospital stay.

**Results:**

Two patients in the EOR group experienced pain relapse (3.2%), and four patients in the UOR group experienced pain relapse (6.77%) after oral refeeding (*p* = 0.379). The presence of nausea or vomiting after the onset of oral refeeding was not different between the two groups (*p* = 0.293). The onset of oral refeeding was approximately 48 h later in the UOR group. The length of hospital stay was 5 days in the EOR group and 8 days in the UOR group (*p* = 0.042), and this difference was also manifested in higher hospital costs in the UOR group (*p* = 0.0235).

**Conclusion:**

Compared with usual oral refeeding, early oral refeeding is safe in predicted mild acute biliary pancreatitis patients, does not cause adverse gastrointestinal events, and reduces the length of hospital stay and costs.

**Trial registration:**

Early oral refeeding in mild acute pancreatitis (EORVsUOR). NCT04168801, retrospectively registered (November 19, 2019).

## Background

Acute pancreatitis (AP) is the result of an inflammatory process in the pancreas, and although the causes are varied, the pathophysiology and management are similar. AP is most commonly caused by bile stones, presents with different degrees of severity, and causes local and systemic complications, leading to high catabolic, hypermetabolic, and hyperdynamic stress states [1]. The currently widely used severity assessment tool used is the revised Atlanta classification, wherein AP is classified as mild, moderate and severe [2]. As no curative therapy is currently available for AP, early treatment consists of supportive care, which includes adequate fluid resuscitation, pain management and enteral nutrition [3]. Nutrition therapy is an essential component of AP management [4]. The published IAP/APA guidelines recommended that oral feeding in patients with predicted mild pancreatitis can be restarted once abdominal pain has decreased and the levels of inflammatory markers have improved (Recommendation G. Nutritional support 20-Grade 2B, strong agreement) [5]. This recommendation is reinforced by other authors with similar clinical results [6, 7]. Pancreatic rest by the nil per os (NPO) strategy has been considered necessary in patients with AP until the resolution of abdominal pain and decreases in the levels of pancreatic and inflammatory markers [8]. This trend has changed; now, it is clear that early oral refeeding (OR) for mild PA not only provides adequate caloric intake but also may improve clinical outcomes. It has been hypothesized that the combination of disturbed intestinal motility, microbial overgrowth and increased permeability of the gut can lead to bacterial translocation, thus causing infection and pancreatic necrosis [9, 10].

OR may reduce translocation by stimulating intestinal motility and reducing bacterial overgrowth, thereby maintaining mucosal gut integrity [11, 12]. Additionally, infection complications, organ failure and mortality are decreased in patients receiving early oral refeeding compared with those receiving routine total parenteral nutrition [13, 14]. In patients with predicted mild AP, numerous studies have shown that a normal oral diet can be resumed once the pain has started to decrease [1, 15–17]. However, it remains unclear what the optimal timing is for OR. There is still no consensus about the definition of “early” refeeding, the criteria for refeeding, the estimated daily energy intake and type intake [18].

The aim of the present study was to compare the early onset of OR (within the first 24 h after hospital admission) with usual OR (25–72 h after hospital admission) and determine whether the early introduction of OR influences the recurrence of pain or alters the blood levels of pancreatic enzymes in patients with mild acute pancreatitis.

## Methods

### Patients

This randomized controlled trial was carried out between September 2018 and June 2019 after authorization from the ethics committee for health research. All patients admitted to the Surgery Department with a diagnosis of biliary acute pancreatitis (BAP), with a predicted mild episode severity based on the criteria and a symptom onset time less than 24 h, were screened for inclusion in the study. Patients with pancreatitis stemming from another cause, (i.e., pregnancy, history of chronic pancreatitis), those under 18 or over 75 years, and those with predicted moderately severe or severe acute biliary pancreatitis were excluded. Written informed consent was signed from all patients.

### Sample size

A total of 124 patients were randomized in this study. The sample size was calculated according to the formula published by Bouman et al. 2015 [19], in which a percentage of success of 90% was estimated with the standard treatment compared to 85% for the experimental management, with a margin of no less than 5%, an alpha of 0.05%, a beta of 20%, and a percentage of estimated losses of approximately 10%, for a total of 62 patients per group.

### Definitions

The diagnosis of AP was established when the patient presented two or more of the three following findings: typical abdominal pain, elevation of serum pancreatic enzymes (amylase and/or lipase) more than three times the upper limit of normal, and imaging study (ultrasonography or computed tomography) results suggestive of AP [20]. On admission, the *etiology* of acute pancreatitis was determined using detailed personal (i.e., previous acute pancreatitis, known gallstone disease, alcohol intake, medication and drug intake, known hyperlipidemia, trauma, recent invasive procedures such as endoscopic retrograde cholangiopancreatography (ERCP)) and family histories of pancreatic disease, physical examinations, laboratory serum tests (liver enzymes, calcium, triglycerides), and imaging (right upper quadrant ultrasonography). Patients with acute pancreatitis from a cause other than biliary pancreatitis were excluded [5].

### Severity assessment

The severity assessment of AP was performed based on the revised Atlanta classification, and the severity was classified as mild, moderately severe and severe. In the absence of organ failure and local or systemic complications, AP was labeled as predicted mild; it was defined using the modified Marshall scoring system [21], and only the patients with complete information for the determination of the severity of AP were included in this study.

### Protocol

Once the diagnosis of acute biliary pancreatitis was confirmed and the course was predicted to be mild, written informed consent was obtained.

Patients were divided into Group A (early oral refeeding, EOR) and Group B (usual oral refeeding, UOR) through a random number table generated with the commercial statistics program IBM SPSS version 25 (the numbers generated by the program were for the experimental group).

Due to the characteristics of the study, only simple blinding was possible (the doctor who performed the statistical analysis).

Both groups were medically managed according to the IAP and APA guidelines [5].

Fluid therapy with crystalloid solution (Hartmann) was administered at an initial bolus of 10 mL/kg, followed by the infusion of 1.5 mL/kg/h for 24 h.

Pain was managed with the weak opioid tramadol 50 mg every 6 h and paracetamol 1 g every 8 h with continuous evaluation with the numerical analogue scale (NAS) to determine the need for extra doses.

### Oral Refeeding

Group A: In the EOR group, once a patient had a score of 1–3 on the NAS, he was asked about symptoms such as nausea or vomiting; if he did not have such symptoms, then he received OR as indicated between 16 and 24 h after admission.

Group B: In the UOR group, once a patient had a score of 1–3 on the NAS, he was asked about symptoms such as nausea or vomiting; if he did not have such symptoms, then he received OR as indicated between 25 and 72 h after admission.

### Type of diet

In both groups, the initial diet was the same to avoid influencing the outcomes.

The soft diet consisted of one 900 Kcal meal per day, composed of 86.7% carbohydrates (190 g), 13.3% protein (30 g) and 0% lipids (0 g).

When the diet was adequately tolerated and there was no evidence of clinical complications or deterioration, a normal diet was indicated, and the follow-up continued.

### Endpoints

During the entire hospital stay or until surgery, the Marshall scale continued to indicate the classification of AP as mild. None of the patients evaluated had an aggravation of the episode. Follow-up was conducted 2 months postsurgery.

The outcome measures were the levels of the pancreas-specific markers amylase and lipase, the systemic inflammatory response (concentrations of leukocytes), feasibility (evaluated by abdominal pain recurrence), the presence and recurrence of gastrointestinal symptoms and the length of hospital stay.

### Data collection

Laboratory data, such as the levels of leukocytes, amylase and lipase, were collected after inclusion in the study and after 24 and 48 h of oral refeeding. Clinical data included age, sex, time from onset of pain (baseline), Marshall scores at admission and after starting oral feeding, gastrointestinal symptoms, abdominal pain, days until solid food intake, pain relapses, complications, length of hospital stay and readmission.

### Statistical analysis

Data are presented as frequencies and percentages, and comparisons between groups were performed using the χ^2^ test for binary data or Fisher’s exact test. Continuous variables are presented as the median and range or interquartile range and were compared using the Mann-Whitney U-test or Student’s t test if they met the criteria for a normal distribution. *P*-values less than 0.05 were considered significant. Statistical analyses were performed with SPSS version 25.0.0. Analysis by intention to treat was used.

## Results

**Fig. 1:**
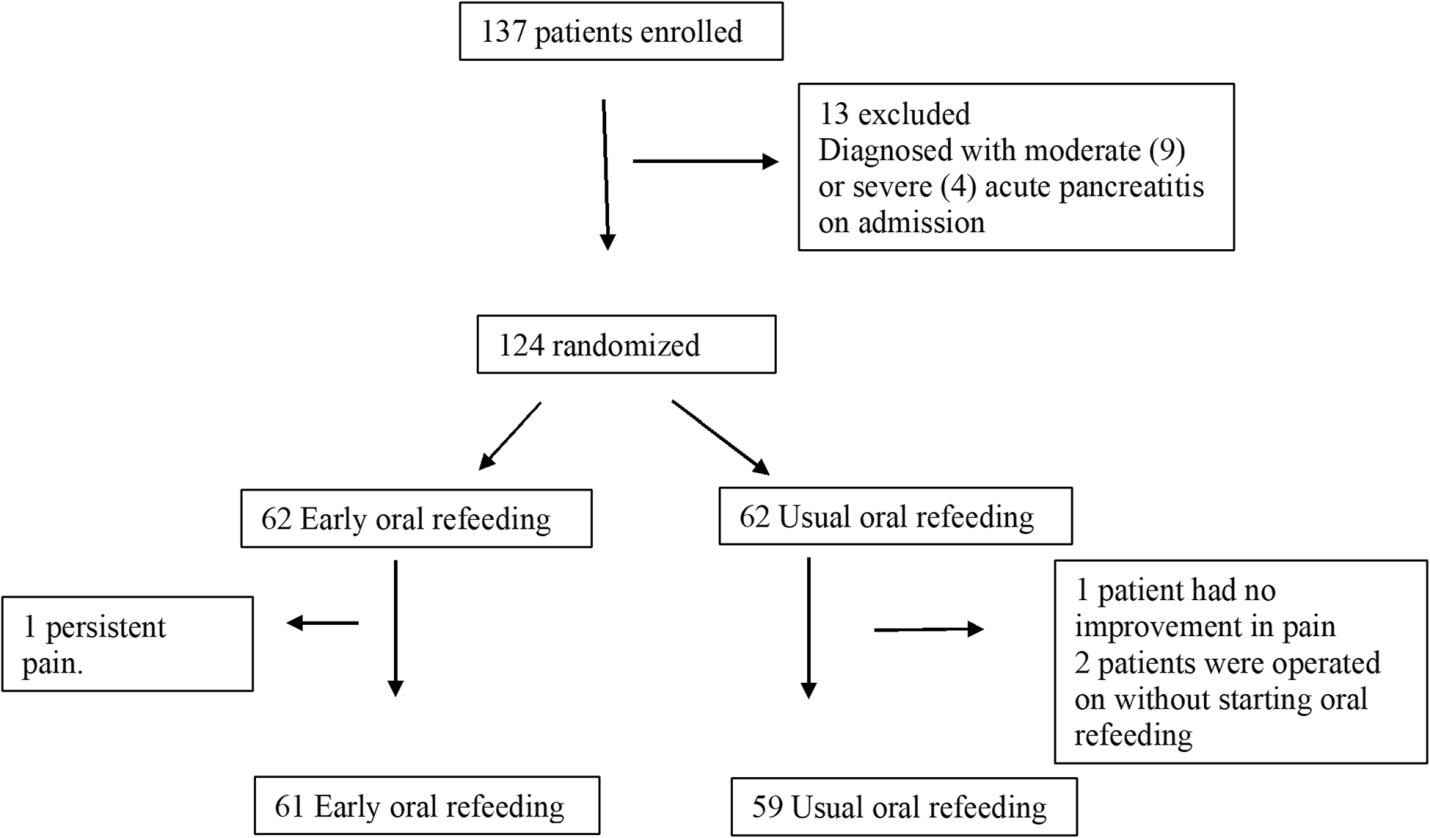
CONSORT flow diagram

A total of 124 patients were randomized, and 120 were included (61 in the EOR group and 59 in the UOR group). As shown in Fig. 1 (CONSORT diagram); one patient from the EOR group was excluded because of persistent pain and his consequent inability to receive oral refeeding. Three patients in the UOR group were excluded because one patient had no improvement in pain; computed tomography was performed, and peripancreatic collections were identified. Two patients were operated on without starting OR. Fig. 1. These four patients who were excluded from the study represent a loss of 3.2% of the study population.

The demographic data and clinical parameters of patients at admission are presented in Table 1. There were no statistically significant differences between the two groups.

**Table 1:** Demographic data and clinical parameters on admission

Variable	EOR *n* = 61	UOR *n* = 59	p
Age (years)	45.3 (17.02)	50.01 (14.2)	0.106^a^
Sex (female/male)	46/15	41/18	0.271^b^
Weight (kg)	73.2 (11.08)	71.9 (9.58)	0.499^a^
Height (meters)	1.61 (0.06)	1.58 (0.03)	0.969^a^
BMI (kg/m^2^)	28.04 (3.87)	27.6 (4.67)	0.531^a^

^a^ Continuous variables are presented as the mean and standard deviation and were compared using Student’s t test

^b^ Data are presented as frequencies, and comparisons between groups were performed using the χ^2^ test

The comparisons of outcome variables between the two groups are presented in Table 2. Two patients in the EOR group experienced pain relapse (3.2%), and four patients in the UOR group experienced pain relapse (6.77%) after OR (*p* = 0.379). Another characteristic that determines tolerance of OR is the presence of gastrointestinal symptoms; there was no difference in the occurrence of nausea or vomiting after the onset of OR between the two groups (*p* = 0.293).

**Table 2:** Comparison of outcome variables between the two groups

Variable	EOR *n =* 61	UOR *n =* 59	p
Lipase (0 h) IU/L	547.1 (245–760)	530 (240–840)	0.644^a^
Lipase IU/L after oral refeding	485 (174–530)	110 (85–200)	0.665^a^
Leukocytes (0 h) 10^3^UL	10.2 (7.29–13.7)	10.3 (7.8–14)	0.622^a^
Leukocytes after oral refeeding 10^3^UL	10.8 (7–13.2)	7.2 (7–13.2)	0.658^a^
Paín (NAS) (0 h)	9 (6–10)	8 (7–10)	0.812^a^
Pain (NAS) After oral refeeding	3 (0–3)	3 (1–4)	0.842^a^
Time of evolution(h)	20 (12–24)	23 (14–23)	0.668^a^
Nausea or vomiting (0 h)	39 (63.9%)	45 (76.2%)	0.186^b^
Nausea or vomiting after oral refeeding	1 (1.6%)	3 (5%)	0.293^b^
Pain relapse	2 (3.2%)	4 (6.77%)	0.379^b^

^a^Continuous variables are presented as the medians and interquartile range and were compared using the Mann-Whitney U-test

^b^ Data are presented as frequencies and percentages, and comparisons between groups were performed using the χ^2^ test

The serum lipase level could indicate recurrence; in the EOR group, the serum lipase level did not increase after the onset of OR compared to the level at admission. In the UOR group, a decrease in the serum lipase level was observed after the start of OR compared with the baseline level at admission; however, this finding was expected and was in fact an inclusion criterion for this group.

The systemic inflammatory response was evaluated based on the leukocyte levels, and the behavior was very similar to that described for the lipase levels. The levels in the EOR group did not increase after the onset of the OR compared to the level at admission.

The length of hospital stay and follow-up are presented in Table 3. The onset time of OR was approximately 48 h longer in the UOR group. The length of stay in the hospital was 5 days in the EOR group and 8 days in the UOR group (*p* = 0.042), and this difference was also manifested in higher hospital costs in the UOR group (*p* = 0.0235).

**Table 3:** Hospital length of stay comparison and follow up

Variable	EOR *n =* 61	UOR *n =* 59	p
Time for start OR	18 (12–22)	72 (32–96)	0.001^a^
Hospital stay length (days)	5 (3–8)	8 (7–10)	0.032^a^
Hospital cost (dollars)	2089 (2006–3812)	3310 (2810–4019)	0.0235^a^

^a^Continuous variables are presented as the medians and interquartile ranges and were compared using the Mann-Whitney U-test

## Discussion

For decades, pancreatic rest by the nil per os (NPO) strategy was considered necessary for patients with AP until the abdominal pain resolved and the levels of pancreatic and inflammatory markers normalized. This trend has changed now; early enteral feeding is accepted in the treatment of AP, but there is still no consensus about the definition of early oral refeeding.

The concept of early oral refeeding includes the time between admission and the start of the diet and the presence of adverse events, including abdominal pain relapse and gastrointestinal symptoms (nausea and vomiting).

In this study, we found that EOR is safe within the first 24 h after hospital admission and that there is no difference in the presence of abdominal pain relapse, nausea or vomiting compared with standard oral refeeding in patients with predicted mild acute biliary pancreatitis. This study provide solid evidence for the oral intake should be started within 24 h.

The criteria for EOR are unclear. Eckerwall GE 2007 [15] reported that his patients were “immediately allowed to drink”, and the diet start time was 1 day. Teich N 2010 [17] did not report a criterion; the onset time was determined by the randomization, and the start time for his patients was 2 days. Li J 2013 [1] reported the “feeling of hunger” as the criterion for the start of OR, and the start time for his patients was 5 days. Larino-Noia 2014 [22] reported “normal bowel sounds” as the criterion for starting OR, and the diet start time for his patients was 2 days. Our criteria were the objective measurement of symptoms, a pain score of 3–10 on the NAS, and the absence of symptoms such as nausea or vomiting. These criteria allowed OR to begin within the first 24 h, with a 95% success rate.

Once the start time for OR is defined, the next point to clarify is the type of diet. The meta-analysis conducted by Meng et al. in 2011 [23] showed that in comparison with a clear liquid diet, early OR with a solid diet might provide better outcomes and is safe for patients with AP. Based on these results, we started with a solid diet in both groups, so the diet type did not influence the results of the study; the results show that this type of diet can be started without complications.

In a systemic review and meta-analysis, Horibe M et al. 2016 [24] reported that early OR reduces the length of hospital stay without significant differences in adverse events. In our study, the hospital stay was shorter in the EOR group than in the UOR group (5 vs 8 days) (*p* = 0.042), which also led to lower hospital costs in the EOR group (2089 vs 3310 dollars) (*p* = 0.0235). This cost represents the total hospital stay expenses. The results of this study might impact the treatment strategy and potentially reduce the cost of hospitalization for these patients.

Eckerwall GE 2007 [15] analyzed recurrence and the systemic inflammatory response, measured the pancreatic-specific serum amylase levels and CRP concentrations, and observed any significant differences between groups in any of those biochemical markers for any days evaluated. In our study, we measured serum lipase levels and leukocytes levels as parameters of recurrence and the systemic inflammatory response; we found that the timing of the onset of OR did not influence these parameters, indicating that it did not affect the natural history of the disease.

Horibe M 2020 [25] analyzed the benefits and safety of the immediate oral intake of low-fat solid food in patients with mild AP who were allowed to use opioid analgesics. They found that the beginning of the diet is safe and reduces the cost and the days of hospitalization. Our findings were similar, however we did not use fats, used it in the same way in the control group and we did not use opioids as the main analgesic, so the only difference point between both groups was the beginning of the diet.

The strengths of this study were that all patients had the same cause of AP (biliary), the disease had evolved for at least 24 h in all patients, and all patients received the same type of diet, increasing the homogeneity of the groups. Another strength is the clear and objective criteria for starting the diet. A follow-up visit was conducted 2 months postsurgery to determine the recurrence of disease.

A limitation of the present study is that the design did not include blinding. The nature of the intervention (EOR vs UOR) makes the group allocation obvious, and the patients and medical staff were aware of the classifications. Another limitation was the use of leukocytes and not CRP as a marker of the systemic inflammatory response.

## Conclusion

Early OR is safe in patients with predicted mild acute biliary pancreatitis without adverse gastrointestinal events; in addition, it reduces the length of hospital stay and cost compared with usual OR.

## Data Availability

The datasets during and/or analysed during the current study available from the corresponding author on reasonable request.

## Abbreviations

AP: Acute pancreatitis
OR: Oral refeeding
EOR: Early oral refeeding
UOR: Usual oral refeeding
NAS: Numerical Analogue Scale
NPO: Nil per os
